# Repeated intramuscular transplantations of hUCB-MSCs improves motor function and survival in the SOD1 G^93^A mice through activation of AMPK

**DOI:** 10.1038/s41598-020-58221-1

**Published:** 2020-01-31

**Authors:** Myung Geun Kook, SeungEun Lee, Nari Shin, Dasom Kong, Da-Hyun Kim, Min-Soo Kim, Hyun Kyoung Kang, Soon Won Choi, Kyung-Sun Kang

**Affiliations:** Adult Stem Cell Research Center and Research Institute for Veterinary Science, College of Veterinary Medicine, Seoul National University, Seoul, 08826 Republic of Korea

**Keywords:** Amyotrophic lateral sclerosis, Mesenchymal stem cells

## Abstract

Amyotrophic lateral sclerosis (ALS) is a fatal neurodegenerative disease that is characterized by loss of motor neurons and degeneration of neuromuscular junctions. To improve disease progression, previous studies have suggested many options that have shown beneficial effects in diseases, especially stem cell therapy. In this study, we used repeated intramuscular transplantation of human umbilical cord blood-derived mesenchymal stem cells (hUCB-MSCs) and observed positive effects on muscle atrophy and oxidative stress. In an *in vivo* study, motor function, body weight and survival rate were assessed, and skeletal muscle tissues were analyzed by western blotting and immunohistochemistry. After intramuscular transplantation, the hUCB-MSCs survived within the skeletal muscle for at least 1 week. Transplantation ameliorated muscle atrophy and the rate of neuromuscular degeneration in skeletal muscle through reductions in intracellular ROS levels. Both expression of skeletal muscle atrophy markers, muscle atrophy F-box (MAFbx)/atrogin1 and muscle RING finger 1 (MuRF1), were also reduced; however, the reductions were not significant. Moreover, transplantation of hUCB-MSCs improved protein synthesis and inhibited the iNOS/NO signaling pathway through AMPK activation. Our results suggest that repeated intramuscular transplantation of hUCB-MSCs can be a practical option for stem cell therapy for ALS.

## Introduction

Amyotrophic lateral sclerosis (ALS) is a fatal neurodegenerative disease that leads to the motor neuron degeneration, muscle atrophy and paralysis. Approximately 90% of ALS cases are sporadic (sALS), and 10% of ALS cases are dominantly inherited as familial ALS (fALS) caused by large numbers of mutations and variants in genetic factors^1^. Despite scientific development, the cause of ALS remains mostly unknown, and the approved treatment options have limited effects on disease progression and survival^2^. One of the reasons for the difficulty in investigating and developing therapeutic agents is the complexity of the non-cell-autonomous status of the disease^3^. During ALS disease progression, the loss of neuromuscular junction and muscle atrophy are critically related to disease progression^4^. However, most studies have focused on the central nervous system, especially on the motor neurons, as the main targets to improve disease progression and survival^5–7^. As mentioned above, due to the dynamic communication of various cells involved in disease progression, such approaches that only focus on motor neuron death are not effective for developing therapeutic agents.

In skeletal muscle of ALS, skeletal muscle atrophy is a process that underlies pathological condition of ALS. Previous studies have shown that several factors are involved in skeletal muscle atrophy and one of the best studied molecule is the transforming growth factor type-β (TGF-β). Especially, TGF-β1 level was elevated in the serum and plasma of ALS patients^8^. Also, other studies showed that the TGF-β family were significantly increased in human and mice ALS muscle tissue and suggested that TGF-β family could be strong marker of disease progression and onset^9,10^. Especially, oxidative stress induced by TGF-β1 in the skeletal muscle is essential feature of ALS muscle pathology^11^. These results showed that TGF-β1 and oxidative stress in skeletal muscle closely is related with disease progression and could be therapeutic target in severe skeletal muscle degeneration of ALS. Thus, investigating the pathological mechanism of skeletal muscle of ALS is important for ALS disease. However, how skeletal muscle contributes to disease progression remains unclear, and methods with which to ameliorate disease pathology by targeting skeletal muscle have not been well established to date.

Many studies have investigated the mechanisms of motor neuron death in ALS disease. Among them, AMPK signaling has been recently suggested as a therapeutic target in ALS. During disease progression, Oxidative stress dysregulated AMPK signaling and induced pathological phenotype in ALS^12–15^. Several studies have modulated AMPK activity for ALS therapy, but the results of these studies have been controversial. Metformin, which activates AMPK signaling, accelerates disease progression in hSOD1-G^93^A mice^16^. Another study also confirmed that inhibition of AMPK signaling is beneficial *in vitro* and in *C. elegans* ALS models^12^. In contrast, latrepirdine and resveratrol, which are small-molecule activators of AMPK, have been shown to improve lifespan and delay disease onset in hSOD1-G^93^A mice^6,17^. Samuel and colleagues also provided evidence that AMPK activation alleviates synaptic dysfunction of the outer retina in aged mice through synaptic remodeling^18,19^. Overall, although it is unclear whether activation of AMPK signaling is harmful or beneficial in ALS, previous studies have suggested that AMPK signaling is an important molecular target for therapeutic strategies for ALS.

Although many previous studies have investigated therapeutic agents for ALS, a successful treatment for ALS has not been found. However, stem cell therapy, which uses cells that characteristically release different growth and trophic factors that are crucial for the survival of damaged tissue and cells, is a promising option for the treatment of neurodegenerative disease^20,21^. In particular, in recent clinical trials, intrathecal and intramuscular administration of human mesenchymal stem cells (hMSCs) in patients with ALS were found to be safe and to provide beneficial effects for patients, including up to 25% improvement in the slope of progression. Furthermore, another study showed that repeated intrathecal administration of MSCs showed a possible clinical effect for at least 6 months in ALS patients^7,20–23^. Therefore, application of hMSCs could lead to the generation of beneficial effects for neurodegenerative diseases.

In this study, we performed repeated intramuscular transplantation of hUCB-MSCs in hSOD1-G^93^A mice. In a previous study, hMSC application through intramuscular injection was found to elicit positive results in hSOD1-G^93^A mice^20,21^, but how intramuscular transplantation of hUCB-MSCs improves the progression of disease is unknown. Therefore, we investigated the therapeutic mechanisms of hUCB-MSCs with regard to modulation of muscle atrophy and AMPK activation in the C2C12 myotube and skeletal muscles of hSOD1-G^93^A mice.

## Results

### TGF-β1 induced muscle atrophy was ameliorated by hUCB-MSCs

**Figure 1:**
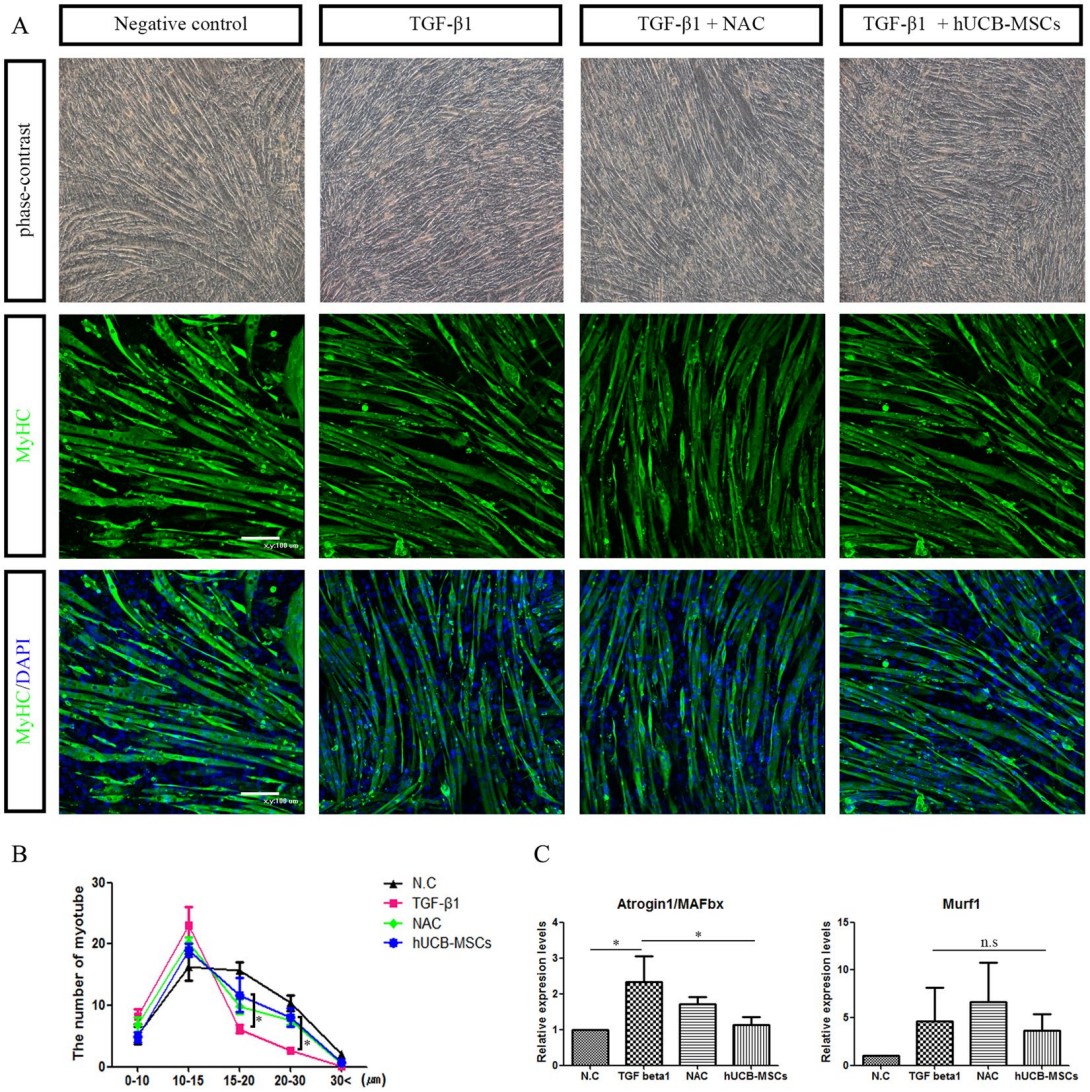
Human umbilical cord blood-derived mesenchymal stem cells (hUCB-MSCs) prevent TGF-β1-induced muscle atrophy. (**A**) Phase-contrast and immunofluorescence images of myotubes treated with or without TGF-β1 (10 ng/ml) for 24 hr in the coculture with hUCB-MSCs. (**B**) The number of myotubes for each width was measured and plotted as the graph. TGF-β1 vs hUCB-MSCs. *P < 0.05. (**C**) mRNA levels of muscle atrophy marker, MAFbx/atrogin1 and Murf1, were measured using real-time PCR. Values are standardized to the GADPH housekeeping gene and normalized relative to negative control. All data were analyzed by one-way analysis. *P < 0.05, n.s: not significant.

A previous study suggested that mesenchymal stem cell-conditioned media reduced muscle atrophy-related proteins in C2C12 cells^11,24,25^. To confirm whether hUCB-MSCs could prevent TGF-β1 induced muscle atrophy in C2C12 cell myotubes, we performed hUCB-MSCs coculture study in C2C12 cell myotubes treated with TGF-β1 and measured diameter in each group of C2C12 cell myotubes 24 hr after TGF-β1 treatment. We found that TGF-β1 treatment increased the number of low width C2C12 cell myotubes compared to control group (0–10 μm; TGF-β1: 8.14, Control: 5.13, 10–15 μm TGF-β1: 23.0, Control: 16.3) and hUCB-MSCs group ameliorated decrease of the number of high width C2C12 cell myotubes compared to the TGF-β1 treated group (P < 0.05, 15–20 μm TGF-β1: 6.14, hUCB-MSCs: 11.7, 20–30 μm TGF-β1: 2.71, hUCB-MSCs: 8.00). Pretreatment of NAC protected the C2C12 cell myotubes from TGF-β1 treatment (20–30 μm TGF-β1: 2.71, NAC: 7.63; Fig. 1A,B). Although mRNA level of MuRF1 which is representative muscle atropy marker was not influenced, we observed that mRNA level of MAFbx/atrogin1 which is another muscle atrophy marker was significantly decreased in hUCB-MSCs group (P < 0.05, Fig. 1C). Taken together, these result demonstrated that coculture of hUCB-MSCs prevented the muscle atrophy induced by TGF-β1.

### Direct or indirect coculture of hUCB-MSCs prevented muscle atrophy in C2C12 myoblast cells by reducing ROS

**Figure 2:**
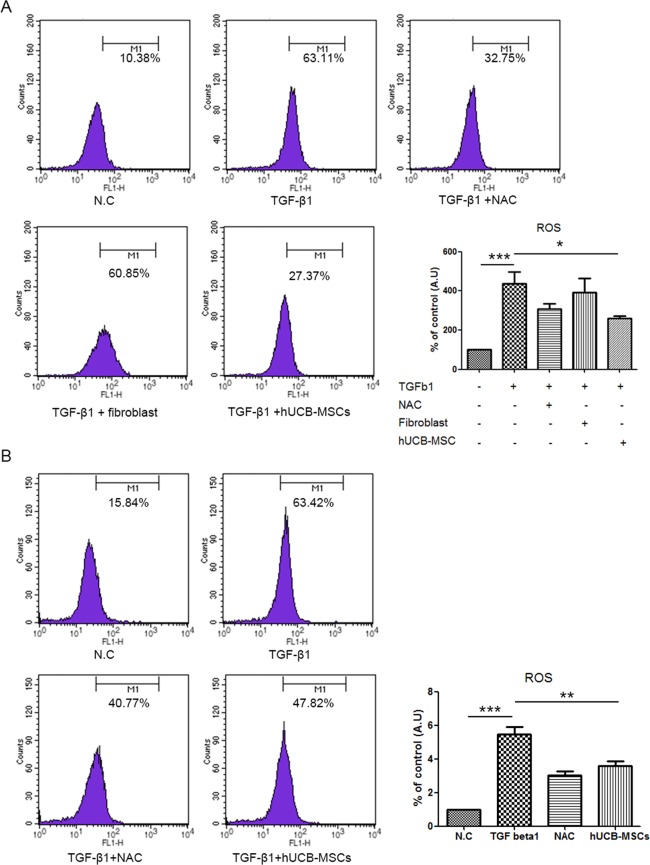
hUCB-MSCs reduced ROS levels induced by TGF-β1 in C2C12 cell myoblasts. (**A**) C2C12 myoblast cells were directly cocultured with hUCB-MSCs with or without TGF-β1. Cocultured myoblast cells were stained with H_2_DCFDA to measure intracellular hydrogen peroxide using FACS caliber. (**B**) To confirm indirect effect of hUCB-MSCs, TGF-β1 treated C2C12 myoblast cells were cocultured with or without hUCB-MSCs and measured intracellular hydrogen peroxide levels. Graphs were plotted as the percentage relative to negative control and analyzed by one-way analysis. *P < 0.05, **P < 0.01, ***P < 0.001.

In a previous study, it was demonstrated that TGF-β1 induced muscle atrophy dependent on NOX-induced ROS in skeletal muscle^11^. Therefore, we first investigated whether hUCB-MSCs could inhibit ROS in C2C12 myoblast cells. Similar to the findings in a previous study, hUCB-MSCs effectively reduced ROS levels induced by oxidizing agent tert-butyl hydroperoxide (tbH_2_O_2_) in C2C12 myoblast cells (P < 0.001). Additionally, the expression of MAFbx/atrogin1 mRNA, but not MuRF1 mRNA, was significantly reduced in the hUCB-MSC group compared with the tbH_2_O_2_-treated group (P < 0.05, Supplement Fig. S1). Next, we investigated whether C2C12 myoblast cells were protected from muscle atrophy in coculture with hUCB-MSCs through modulation of intracellular ROS levels. As expected, TGF-β1 significantly increased intracellular ROS levels in C2C12 myoblast cells after 24 hr of treatment (Supplement Fig. S2), and we also found that direct coculture of hUCB-MSCs reduced intracellular ROS levels in C2C12 myoblast cells (Fig. 2A). Furthermore, a previous study found that umbilical cord-mesenchymal stem cell (UC-MSC)-conditioned media could prevent oxidative stress in atrophied muscle cells by recovering the expression of antioxidant enzymes^26^. To confirm whether hUCB-MSCs could indirectly modulate the ROS levels induced by TGF-β1, we performed indirect coculture with hUCB-MSCs. Again, hUCB-MSCs could suppress the generation of intracellular ROS in C2C12 myoblast cells through an indirect method (Fig. 2B).

### Indirect co-culture of hUCB-MSCs regulated inducible nitric oxide synthase (iNOS) and protein synthesis through activation of AMPK

**Figure 3:**
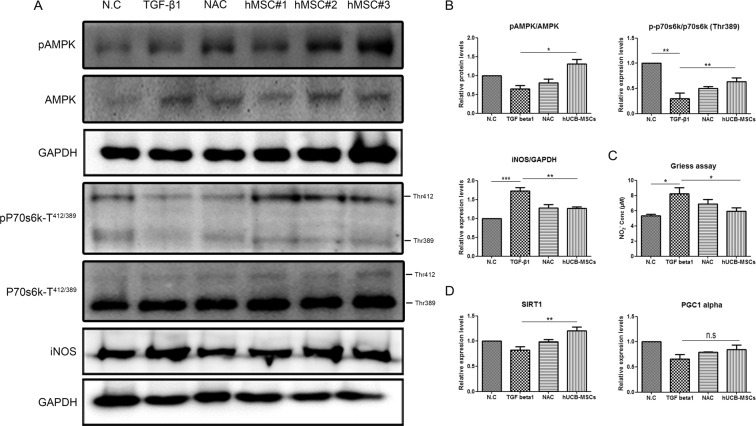
Activation of AMPK by hUCB-MSCs regulated iNOS and protein synthesis in C2C12 cell myotubes. (**A**) C2C12 cell myotubes were treated with TGF-β1 for 24 hr and cocultured with or without hUCB-MSCs and the cell lysates were probed with the indicated antibody. (**B**) The bands of (**A**) were quantified, and the relative expression levels are shown in the graph. (**C**) NO concentration in myotubes treated as described in (**A**) was measured using the Griess assay. (**D**) Representative graph showed relative change in the mRNA expression of SIRT1 and PGC1-alpha. All data were represented as mean ± SEM for three independent experiments and were analyzed by one-way analysis. *P < 0.05, **P < 0.01, ***P < 0.001.

Intracellular ROS has been suggested to regulate mitochondrial biogenesis in many cell types. In particular, SIRT1/AMPK/PGC1 alpha is a critical regulator of mitochondrial biogenesis^27^. Therefore, we investigated whether the modulation of intracellular ROS by hUCB-MSCs is related to mitochondrial dysfunction induced by TGF-β1 in C2C12 cell myotubes. To determine which components of mitochondrial dynamics were mediated, we assessed the protein levels of AMPK in C2C12 cell myotubes. TGF-β1 alone did not activate AMPK in C2C12 cell myotubes, but hUCB-MSCs induced AMPK activation (Fig. 3A). SIRT1 and PGC1-alpha, which are related to the AMPK pathway, were increased in the hUCB-MSC group, but the increase in PGC1-alpha was not significant (Fig. 3D). Next, we performed real-time PCR analysis of the mitochondrial dynamics-related genes mitofusin1 (Mfn1), mitofusin2 (Mfn2), optic atrophy protein1 (OPA1), dynamin-related protein1 (DRP1) and BCL2/adenovirus E1B 19 kDa protein-interacting protein 3 (Bnip3), but the expression of mitochondrial dynamics-related genes was not significantly different among the groups of C2C12 cell myotubes (Supplement Fig. S3).

To investigate other components by which hUCB-MSCs could prevent muscle atrophy, we investigated iNOS and protein synthesis levels in C2C12 cell myotubes. Each line of hUCB-MSCs reduced iNOS protein levels, corresponding with the decreased levels of NO in the indirect coculture with hUCB-MSCs (Fig. 3A,C). Furthermore, increases in oxidative stress and iNOS protein levels have been implicated in anabolic signaling inhibition in muscle wasting^28^. Therefore, we assessed the protein level of ribosomal protein S6 kinase (p70S6K). Oxidative stress induced by TGF-β1 reduced the phosphorylation level of the p70S6K protein after 24 hr of treatment, and both NAC and hUCB-MSCs prevented the reduction in the phosphorylation level of the p70S6K protein (Fig. 3A,B).

### Repeated intramuscular application of hUCB-MSCs improved motor function and extended survival of hSOD1-G^93^A mice

**Figure 4:**
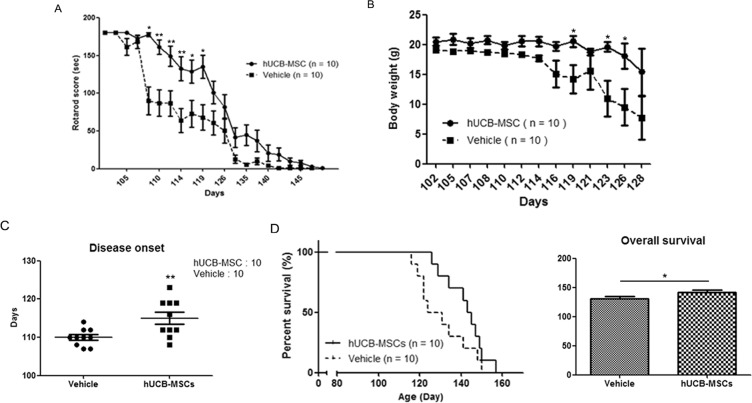
Repeated intramuscular transplantation of hUCB-MSCs improved the motor function and survival of hSOD1-G^93^A mice. The motor functions of mice were assessed using (**A**) rotarod, (**B**) body weight. (**A**) The mice that were repeatedly injected with hUCB-MSCs into gastrocnemius muscle showed a significant improvement in the rotarod test. (**B**) The loss of body weigh was delayed in hUCB-MSCs group in comparison with the vehicle treated group. (**C**) Disease onset was improved in the hUCB-MSCs group. (**D**) The lifespan of mice was prolonged by hUCB-MSCs when compared with the vehicle group. *P < 0.05, **P < 0.01.

To evaluate the potential use of hUCB-MSCs in hSOD1-G^93^A mice as a therapeutic agent, we monitored motor activity through a rotarod test. Rotarod tests were performed every other day, and mice from each group were trained for 1 week before the first intramuscular application of hUCB-MSCs. We found that repeated intramuscular application of hUCB-MSCs significantly improved motor activity from days 108 to 119 (Fig. 4A). Weight loss is a frequent feature in ALS disease. Therefore, we assessed the body weight of each group from days 102 to 128 and found that the change in body weight was significantly different between the groups transplanted with PBS and hUCB-MSCs from days 119 to 126 (Fig. 4B). Disease onset was measured as the time point when the body weight loss of the animal reached 5–6%. The score of the hSOD1-G^93^A mice was improved in the hUCB-MSC-transplanted group (mean 131.7 ± 2.86, p < 0.05) compared with the PBS-injected group (mean 121.7 ± 3.363) (Fig. 4C).

The group that was transplanted with hUCB-MSCs survived significantly longer than the PBS-injected group. The lifespan of the hUCB-MSC-transplanted animals (p < 0.05, mean 144.0 days) was improved compared with that of the PBS-injected animals (mean 127.5 days) (Fig. 4D,E). These results demonstrated that repeated intramuscular application of hUCB-MSCs effectively improved motor function and prolonged lifespan in hSOD1-G^93^A mice.

### The survival of hUCB-MSCs in the gastrocnemius muscle of hSOD1-G^93^A mice

We transplanted hUCB-MSCs into the gastrocnemius muscles of hSOD1-G^93^A mice once a week. Thereafter, we investigated whether the transplanted hUCB-MSCs could survive in the gastrocnemius muscle for 1 week. To identify the presence of viable transplanted hUCB-MSCs, we transplanted GFP-tagged hUCB-MSCs and stained the gastrocnemius muscle with a human-specific marker for nuclei (HuNu) after 1 week. We found that GFP-tagged hUCB-MSCs that colocalized with HuNu survived between the muscle fibers for at least 1 week in the gastrocnemius muscles of hSOD1-G^93^A mice (Supplement Fig. S4).

### Intramuscular injection of hUCB-MSCs in hSOD1-G^93^A mice ameliorated muscle atrophy

**Figure 5:**
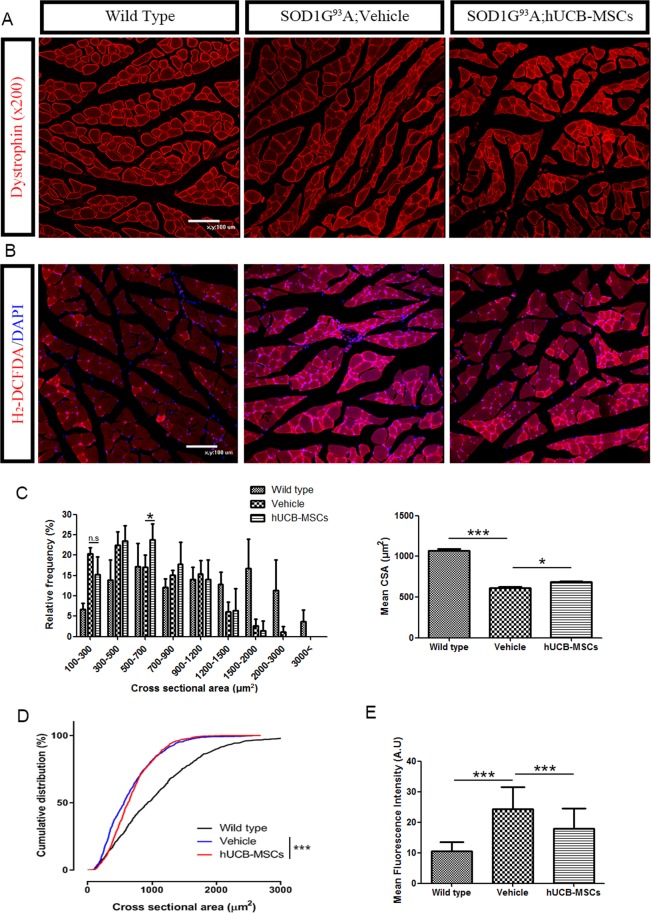
Repeated intramuscular injection of hUCB-MSCs ameliorated muscle atrophy via inhibition of ROS generation in the hSOD1-G^93^A mice. (**A**) Immunostaining of gastrocnemius muscles of mice were used to assess cross-section area of muscle fibers. (**B**) ROS generation in gastrocnemius muscles was measured using DHE assay. Representative image of tissue from three group of mice (n = 3) (**C**) Cross-sectional area analysis of the gastrocnemius muscle from 100 fibers per mouse (n = 3). (**D**) Cumulative distribution of cross-sectional area of the skeletal muscle from each group of mice. (**E**) Mean fluorescence intensity of intracellular superoxide from DHE assay. Differences in the groups were analyzed by one-way analysis and data of cumulative distribution was analyzed by Kolmogorov-Smirnov test. *P < 0.05, **P < 0.01, ***P < 0.001.

To investigate whether repeated intramuscular injection of hUCB-MSCs ameliorated muscle atrophy in hSOD1-G^93^A mice, we performed dystrophin staining of the gastrocnemius muscles of mice that presented atrophied muscle phenotypes in each group (Fig. 5A). For precise comparison between the PBS-injected and hUCB-MSC-transplanted groups, we performed cross-sectional area(CSA) analysis of the gastrocnemius muscle in the different groups. The cross-sectional areas of the gastrocnemius muscles of hSOD1-G^93^A mice were mostly distributed between 100 and 1200 µm^2^, but those of the gastrocnemius muscles of wild-type mice were distributed between 300 and 3000 µm^2^. The proportion of fibers in the 500–700 μm^2^ group was higher in the hUCB-MSC-treated group (p < 0.05, Fig. 5C) than in the vehicle-injected group. In contrast, the percentage of small fibers (100 to 300 µm^2^) was higher in the PBS-injected group than in the hUCB-MSC-transplanted group, but the difference was not significant. Similar to the proportion of fibers, the mean total cross-sectional area was higher in the hUCB-MSC group (p < 0.05, 685.2 ± 13.21) than in the vehicle group (615.6 ± 14.41, Fig. 5C). In the cumulative percentage distribution of the CSA, there was a significant difference between wild-type and hSOD1-G^93^A mice. There were leftward shifts in the CSA distributions for the vehicle (649.477 ± 15.531) and hUCB-MSC groups (685.211 ± 13.21) relative to the wild-type group (1066.49 ± 26.8882). The CSA of hUCB-MSC-treated fibers was also shifted to the right under approximately 1000 µm^2^ compared to that of PBS-treated fibers. These results indicated that hUCB-MSCs effectively ameliorated muscle atrophy in the muscles of hSOD1-G^93^A mice (P < 0.001, Fig. 5D).

In association with muscle atrophy, we investigated ROS accumulation in the gastrocnemius muscles of hSOD1-G^93^A mice. ROS accumulation was detected and quantified using a DHE assay. We observed that ROS in the gastrocnemius muscles of hSOD1-G^93^A mice accumulated to high levels (Fig. 5B). For quantification of ROS, we analyzed the intensity of DHE expression in the gastrocnemius muscle in each group and found that the hUCB-MSCs (17.86 ± 1.201) significantly ameliorated ROS accumulation in gastrocnemius muscle compared with the vehicle (24.32 ± 1.529), (P < 0.001, Fig. 5E).

### hUCB-MSCs ameliorate disease progress through AMPK activation in gastrocnemius muscle of hSOD1-G^93^A mice

**Figure 6:**
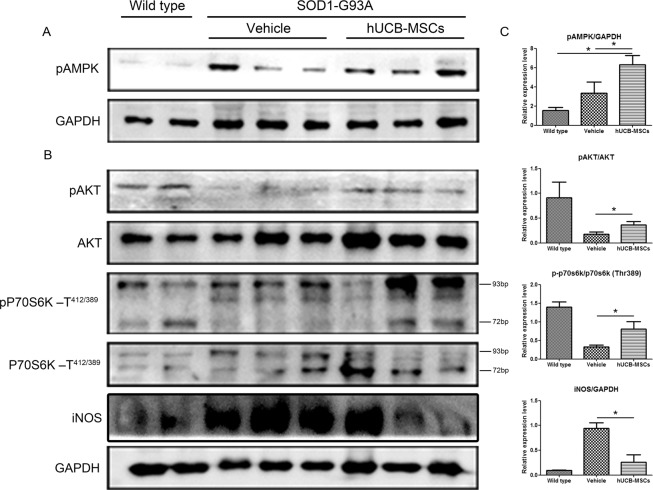
Activation of AMPK regulated protein synthesis and iNOS pathway in the gastrocnemius muscles of hSOD1-G^93^A mice. (**A**) Expression of AMPK increased in the gastrocnemius muscles transplanted with hUCB-MSCs. (**B**) The intramuscular transplantation of hUCB-MSCs rescued expression of phosphorylated AKT and P70S6K proteins and ameliorated iNOS protein expression. (**C**) Statistical analysis of pAMPK, AKT, pP70S6K and iNOS (n = 2~3). *P < 0.05.

To assess whether application of hUCB-MSCs could modulate the iNOS and protein synthesis pathways in the gastrocnemius muscles of hSOD1-G^93^A mice through AMPK activation, as in the *in vitro* studies, we extracted proteins from the gastrocnemius muscles of the mice in each group. We found that transplantation of hUCB-MSCs into gastrocnemius muscle could activate AMPK protein (P < 0.05, Fig. 6A,C). In addition, the phosphorylation of the AKT and p70S6K protein (pP70S6K) expression was significantly decreased in the vehicle group compared to the wild-type group but pAKT and pP70S6K protein level in the gastrocnemius muscles of hSOD1-G^93^A mice was rescued in the hUCB-MSC group compared to the vehicle group (P < 0.05, Fig. 6B,C). Additionally, iNOS protein expression was increased in the muscles of disease mice, and similar to the case in the *in vitro* study, hUCB-MSCs significantly reduced iNOS protein levels (P < 0.05, Fig. 6B,C). These results suggest that hUCB-MSCs modulate protein synthesis and the iNOS pathway, consistent with our *in vitro* results.

### Intramuscular application of hUCB-MSCs ameliorated neuromuscular degeneration in gastrocnemius muscle of hSOD1-G^93^A mice

**Figure 7:**
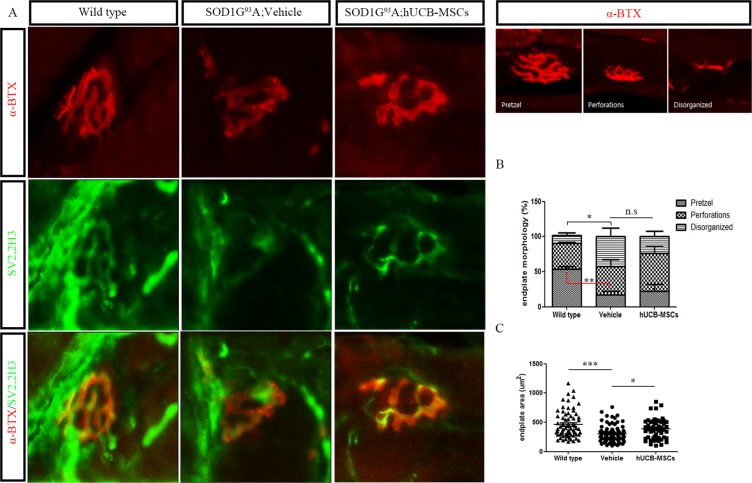
Representative immune-staining and quantitative analysis of neuromuscular junction in the gastrocnemius muscle of hSOD1-G^93^A mice. (**A**) Immunohistochemistry image showed NMJ denervation and rescue of NMJ after transplantation of hUCB-MSCs. (n = 3) (**B**) Endplate morphology was categorized into three type: pretzel, perforated and disorganized. No significant differences between vehicle and hUCB-MSCs groups. (**C**) Measurement of NMJ endplate area of gastrocnemius muscle of hSOD1-G^93^A mice. The transplantation of hUCB-MSCs ameliorated degeneration of endplate. All data represented are means ± SEM and analyzed by one way analysis. *P < 0.05, **P < 0.01, ***P < 0.001.

Oxidative stress inhibition and modulation of AMPK induce beneficial effects in neuromuscular disorder by mediating the maintenance of neuromuscular junctions^19,29^. Thus, we investigated neuromuscular junctions in gastrocnemius muscles to determine whether modulation of AMPK and oxidative stress using hUCB-MSCs ameliorated the degeneration of neuromuscular junctions in the skeletal muscles of hSOD1-G^93^A mice. To visualize denervation of neuromuscular junctions, we used alpha-bungarotoxin, neurofilament (2H3) and synaptic vesicle (SV2) antibodies, and we found that hUCB-MSCs ameliorated denervation between the postsynaptic endplates and presynaptic nerve terminals of the neuromuscular junctions in the gastrocnemius muscles of hSOD1-G^93^A mice (Fig. 7A). To assess the morphology of the neuromuscular junctions in the gastrocnemius muscle, we classified the morphology into one of three types: pretzel, perforated, and disorganized. The wild-type group had a significantly higher level of pretzel morphology (p < 0.01) and low level of disorganized neuromuscular junction (p < 0.05) than the hSOD1-G^93^A mouse group. hUCB-MSC transplantation increased the proportion of perforated neuromuscular junctions and decreased the proportion of disorganized neuromuscular junctions compared to vehicle treatment, but the differences were not significant (Fig. 7B). The endplate area was also increased in the hUCB-MSC group (387.7 ± 23.47, p < 0.05) compared with the vehicle group (304.7 ± 14.24, Fig. 7C). These results suggested that repeated intramuscular application of hUCB-MSCs ameliorated NMJ degeneration in the gastrocnemius muscles of hSOD1-G^93^A mice.

## Discussion

This study showed that repeated transplantation of hUCB-MSCs into the gastrocnemius muscles of hSOD1-G93A mice can be a therapeutic strategy to alleviate disease progression. The hUCB-MSCs effectively improved muscle atrophy and neuromuscular junction while reducing oxidative stress in skeletal muscle and C2C12 myotube cells. These result suggested that the effect of hUCB-MSCs was closely related with modulation of ROS. Interestingly, in the skeletal muscle and C2C12 myotube cells, hUCB-MSCs induced AMPK activation while reducing ROS, thereby suggesting that the AMPK activation that induced by hUCB-MSCs was not related to ROS.

Mesenchymal stem cells have been considered to be potential therapeutic agents for many diseases since they have been shown to have multiple properties, such as the ability to modulate inflammatory conditions and anti-inflammatory conditions and to release different cytokines and growth factors that promote neuroprotection. In particular, in ALS, mesenchymal stem cells have also been used as therapeutic agents to ameliorate disease progression^20^. To date, many studies have shown that mesenchymal stem cells administered into the central nervous system, skeletal muscles or both exert positive effects on lifespan and motor function in disease models^5,7,21^. Although Intramuscular injections of hUCB-MSCs have no effect on survival of motor neurons in the spinal cord of SOD1 transgenic mice (Supplement Fig. S5) like previous study^21^, we could find that hUCB-MSCs have protective effect on skeletal muscle of transgenic mice^25,30,31^. However, the protective mechanisms of hUCB-MSCs in skeletal muscle of ALS disease models are largely unknown. To understand how mesenchymal stem cells prevent muscle wasting and denervation in skeletal muscle during disease progression, we performed repeated intramuscular injection of hUCB-MSCs into hSOD1-G^93^A mice. Interestingly, we found that repeated administration of hUCB-MSCs prevented muscle atrophy in both *in vitro* and *in vivo* models (Figs. 1 and 5). The prevention of muscle atrophy was closely related to modulation of oxidative stress and the iNOS/NO pathway, which is a known effector of muscle wasting. Additionally, we found that hUCB-MSCs increased protein synthesis and AMPK activation in C2C12 cell myotubes and hSOD1-G^93^A mouse skeletal muscles (Figs. 3 and 6). It is speculated that the protective effect of hUCB-MSCs in skeletal muscle might be associated with activation of AMPK and modulation of oxidative stress.

The AMPK activation observed in this study was significantly correlated with protein synthesis. In a previous study, AMPK activation in skeletal muscle induced a different outcome. Activators of AMPK, inflammatory cytokines and metformin, decreased the rate of protein synthesis. However, treatment with AICAR and A-769662, which activate AMPK, restored the levels of phosphorylated S6K and S6 proteins, which are markers of protein synthesis^32^. These findings suggested that AMPK activation induced by AICAR and A-769662 could restore protein synthesis in skeletal muscle. To confirm that the AMPK activation in C2C12 cell myotubes and hSOD1-G^93^A mouse skeletal muscles mediated by hUCB-MSCs was related to protein synthesis, we investigated the protein levels of S6K in C2C12 cell myotubes and hSOD1-G^93^A mouse gastrocnemius muscles and found that hUCB-MSCs restored S6K protein levels in an *in vitro* and *in vivo* study (Figs. 3 and 6). These results suggested that AMPK activation by hUCB-MSCs restored protein synthesis in skeletal muscle, similar to the effects of AICAR and A-769662.

Consistent with this finding, we investigated the iNOS/NO pathway in C2C12 cell myotubes and hSOD1-G^93^A mouse gastrocnemius muscles. TGF-β1, which is one of the pro-cachetic factors, is able to induce muscle wasting through the iNOS/NO pathway. This pathway plays a very important role as a downstream mediator of muscle wasting^33,34^. Additionally, TGF-β1 mRNA and protein expression are significantly increased in skeletal muscles of humans and mice with ALS, and because of this characteristic, TGF-β1 could be used as a muscle biomarker for ALS disease^10^. Previous studies have shown that AMPK activation can prevent muscle wasting induced by inflammation through inhibition of the iNOS/NO pathway^32^. Therefore, we investigated whether hUCB-MSCs could inhibit the iNOS/NO pathway and found that hUCB-MSCs could prevent muscle atrophy by reducing nitrite concentration and iNOS protein levels in *in vitro* and *in vivo* studies (Figs. 3 and 6). To further confirm the effects of hUCB-MSCs on muscle atrophy, we investigated the diameters of C2C12 myotubes and the CSAs of gastrocnemius muscles of hSOD1-G^93^A mice. Our study showed that hUCB-MSCs improved the diameters of C2C12 cell myotubes and the CSAs of the gastrocnemius muscles of hSOD1-G^93^A mice. These results demonstrated that AMPK activation stimulated by hUCB-MSCs could improve muscle regeneration through inhibition of the iNOS/NO pathway and restoration of protein synthesis.

In addition to effects on protein synthesis and inhibition of the iNOS/NO pathway, we found that hUCB-MSCs reduced ROS. In previous studies, intracellular ROS have been found to be essential effectors for stress adaptation and diverse muscle functions^35,36^. However, excessive ROS induce various types of muscular dysfunction, such as inhibition of protein synthesis and muscle wasting. ROS have also been closely associated with various neuromuscular conditions, including aging, Duchenne’s and Becker’s muscular dystrophy and ALS^27,29,37,38^. In this study, we showed that hUCB-MSCs inhibited oxidative stress induced by TGF-β1 in C2C12 myoblast cells and ALS model gastrocnemius muscles (Figs. 2 and 5). Furthermore, hUCB-MSCs could delay the degeneration rate of neuromuscular junctions by ameliorating reductions in endplate areas. This amelioration of the degeneration rate was significantly correlated with reductions in excessive ROS levels. However, the morphology of neuromuscular junctions was not significantly different between the hUCB-MSC group and the vehicle group. The lack of significant difference might be due to the low survival rates of human-origin MSCs or to degeneration of neuromuscular junctions that started in the mice before transplantation of the hUCB-MSCs.

In this report, we showed that intramuscular administration of hUCB-MSCs protected neuromuscular junctions and myotubes through modulation of intracellular ROS levels. Additionally, AMPK activation by hUCB-MSCs increased protein synthesis and inhibited the iNOS/NO pathway in skeletal muscle. These modulations led to prolonged lifespan and increased motor function in hSOD1-G^93^A mice, and the findings suggest that hUCB-MSCs are potential therapeutic agents for ALS.

## Methods

### Animals

The hSOD1 G^93^A transgenic mice (B6.Cg-Tg(SOD1*G93A)1Gur/J) used in this study were purchased from the Jackson Laboratories (USA). All experimental procedures involving animals and their care were conducted in accordance with guidelines of the Seoul National University Institutional Animal Care and Use Committee (SNU IACUC, Dec. 16, 2014) and this study was approved by our local ethic committee “SNU IACUC” (Approval No. SNU-141124–1). Mice were housed in individual cages at a constant environment condition (21–23 °C temperature, 50–60% humidity and 12 hr light/dark cycle) and under specific pathogen-free conditions in the animal facility of Seoul National University. In pre-symptomatic stage, same age, female mice were transplanted with hUCB-MSCs at each experiment. For selection of transgenic mice, genomic DNA was extracted from the tail tip, amplified by PCR and genotyped as described previously^39^. After selection of transgenic mice, 1 × 10^6^ hUCB-MSCs were suspended with normal saline (total volume 20 µl) and intramuscular injected by puncture of the skeletal muscle with a Hamilton syringe (Gauge 27 G, Hamilton, Switzerland) while the needle was slowly retracted to prevent loss of injected cells. For repeated transplantation of cells, we performed first intramuscular injection of hUCB-MSCs at pre-symptomatic stage of mice (12 week old mice). The application of hUCB-MSCs was performed once a week, every same time until the treated mice were 19 week old (8 transplants in total). At predefined time point (132days, 7weeks post-transplant), 3 mice per group were sacrificed for western blot and immunohistochemical analysis. These animals were excluded from motor function and survival studies performed with 10 animals per group.

### Analysis of disease progression

The progression of ALS disease was scored by monitoring body weight and motor function. To assess motor function, we used Rotarod test. Before transplantation of hUCB-MSCs, the animals were received pre-training period for one week and Rotarod test was performed two or three times a week with apparatus (Ugo Basile, Italy). The mice were tested on the Rotarod at a constant speed of 10 rpm over 3 min and tested three times per test as described previously^39^. To determine the onset of disease, we pointed out the day that the mice started to lose body weight up to 5–6% from maximum body weight.

### Cell culture and differentiation

C2C12 cell myoblasts were purchased from ATCC (VA, USA) and were grown in Dulbecco’s modified Eagle’s medium (DMEM, Invitrogen) containing 10% fetal bovine serum (FBS, Gibco). At 80–90% confluence of cells, media was changed to DMEM containing 1% normal horse serum (NHS, Gibco) to differentiate C2C12 cell myoblasts to myotubes.

Isolation, culture and characterization of hUCB-MSCs were performed as described previously^40,41^. hUCB samples were obtained from the Seoul City Boramae medical center cord blood bank (Allcord) and obtained samples were harvested with the mother’s informed consent. The samples were mixed with Hetasep (Stem cell Technologies, Canada) at a ratio of 5:1 and incubated 1 hr at RT. Then, supernatant was collected with Ficoll and centrifuged 2,500 rpm, 20 min for separating mononuclear cells. The isolated cells were seeded at density 2 × 10^5^ cells per cm^2^ on plates in growth media containing D-media (Formula 78–5470EF, Gibco, USA), EGM-2 singlequot and 10% fetal bovine serum (FBS, Gibco, USA). After 3 days, the non-adherent cells were washed out and the adherent cell colonies were cultured and grew to sharp, spindle shaped hUCB-MSCs. This process was performed under regulations and guidelines approved by the Institutional Review board (IRB) of Boramae medical center and Seoul national university (IRB.no 1608/001-021).

### Insert cell coculture of C2C12 cell myotubes and hUCB-MSCs

C2C12 cell myoblasts were differentiated to myotubes in differentiation media following seeded on insults culture dish (SPL, Korea). Thereafter, cells were pretreated or not with either N-acetyl-L-cysteine (NAC, 5 mM) for 2 hr and were treated with transforming growth factor beta 1 (TGF-β1, 10 ng/ml, peprotech) for 24 hr. After incubation, cells were washed twice with phosphate-buffered saline (PBS, Invitrogen) and hUCB-MSCs were cultured on the inserts for 24 hr.

### Immunocytochemistry

C2C12 cell myoblasts were seeded and differentiated on sterilized cover glass circular (SUPERIOR, Germany). C2C12 cell myotubes were then fixed with 4% paraformaldehyde (PFA) in PBS for 10 min and permeabilized with 0.5% Triton X-100 for 10 min at room temperature following washing with PBS. After permeabilization, cells were blocked with 5% normal goat serum (Vector, USA) for 1 hr and incubated with primary antibody against MF20 (1:1000, DSHB) overnight at 4 °C. Thereafter, washing with PBS was performed to remove excessive primary antibody and cells were incubated with appropriated fluorescence secondary antibody (1:1000, Thermo Fisher) for 1 hr. Following incubation with DAPI (1:1000, Santacruz), cells were mounted. For quantification of diameter, the number of 40 to 50 C2C12 cell myotubes were randomly selected per image and measured using image J software (NIH, MD).

### Immunohistochemistry

Isolated gastrocnemius muscles were cryo-embedded with OCT compound (Tissue Tek, Germany) and frozen in liquid nitrogen for cryo-sectioning. Cryo-embedded samples were sectioned into 10-µm serial transverse sections and the sections were transferred to silane coated slide (Muto, Japan). After washing twice with PBS, sections were fixed in 4% cold PFA for 10 min, permeabilized with 0.5% Triton X-100 for 10 min and blocked with 5% bovine serum albumin (BSA, GenDEPOT) for 1 h. After blocking, samples were incubated with primary antibody against dystrophin (1:500, Abcam), ChAT (1:500, Millipore) overnight at 4 °C and then incubated with appropriated fluorescence secondary antibody for 1 hr.

For staining of neuromuscular junctions and nerve terminals, cryo-embedded samples were sectioned into 6-µm serial transverse sections and fixed in 4% cold PFA for 30 min. Sections were incubated with α-Bungarotoxin, Alexa Fluor 594 conjugate (1:500, Invitrogen) for 2 hr at RT and then incubated with primary antibody against SV2, 2H3 (1:100, DSHB) overnight at 4 °C. Fluorescence images were carried out using an Eclipse TE2000 confocal microscope (Nikon, Japan).

### ROS assay

The level of intracellular ROS was determined using 2,7-dichlorofluorescein diacetate (H_2_-DCFDA, Invitrogen). C2C12 cell myoblasts were treated and incubated with H_2_-DCFDA for 20 min at 37 °C in 5% CO_2_. After incubation, Cells were washed twice with PBS and analyzed by flow cytometry. Flow cytometry was performed with a FACS caliber and analyzed using Cell quest software (BD Bioscience, USA). To measure intracellular ROS level in the skeletal muscles, cryo-gastrocnemius samples were sectioned into 10-µm thickness and incubated with DHE (Invitrogen) for 30 min. After washing with PBS, sections were incubated with DAPI (1:1000) and mounted.

### Western blot analysis

Proteins from prepared C2C12 myotube cells and the gastrocnemius muscles of mice were extracted by using PRO-PREPTM (iNtRON, Korea) Protein extraction solution. Western blotting was performed on 10–12% acrylamide gels and blotted on to nitrocellulose membranes. For blocking membranes, membranes were incubated with 5% bovine serum albumin (GenDEPOT, USA) for 1 hr and probed with antibodies with phospho-AMPK (1:1000), AMPK (1:2000), inducible nitric oxide synthase (iNOS, 1:1000), phospho-p70s6k (1:1000), p70s6k (1:1000), phospho-AKT (1:500), AKT(1:1000) and GAPDH (1:2000) for 24 hr at 4 °C. After that, membranes were washed with TRIS-buffered saline with 0.1% tween (TBS-T) and membranes were subsequently probed with the appropriate HRP conjugated secondary antibodies (1:2000) for 1 hr. After washing twice with TBS-T, the blots were exposed to ECL reagent and immunoreactive bands were analyzed using ChemiDOC (FluorChem HD2, Proteinsimple, USA). Data were quantified using Image J 1.38 software (NIH, Bethesda, MD).

### Real-time PCR

Total RNA was isolated from C2C12 samples and the gastrocnemius muscles using TRIzol (INvitron, Waltham, MA) according to the manufacturer’s protocol. The concentration of extracted RNA was quantified by a spectrophotometer (ASP-2680, ACTgene, USA). For cDNA, a reverse transcription was performed using the SuperScript III First-Strand (Invitrogen). The resulting cDNA was used for real-time PCR using SYBR green PCR master mix (Thermofisher). Expression data of the duplicated result were used for 2-ΔΔCt statistical analysis and GADPH expression was used for normalization.

### Quantification and statistical analysis

All statistical analyses were performed in GraphPad Prisms software (version 5). One-way ANOVA followed by Newman-Keuls post hoc test was used to investigate significance between three or more groups. The survival rate of each mice group was determined by the Kaplan-Meier method. All data are presented as the mean ± standard error of the mean (SEM). Significance values were *p < 0.05, **p < 0.01, ***p < 0.001, ns; no significance.

## Supplementary information

Supplementary information .

## Data Availability

All the data and protocol details are available from the corresponding authors upon request.

## References

[1] Riva N, Agosta F, Lunetta C, Filippi M, Quattrini A (2016). Recent advances in amyotrophic lateral sclerosis. J. Neurol..

[2] Lunn JS, Sakowski SA, Feldman EL (2014). Concise review: Stem cell therapies for amyotrophic lateral sclerosis: recent advances and prospects for the future. Stem Cell.

[3] Wang J, Fry CME, Walker CL (2019). Carboxyl-terminal modulator protein regulates Akt signaling during skeletal muscle atrophy *in vitro* and a mouse model of amyotrophic lateral sclerosis. Sci. Rep..

[4] Xiao Y (2018). ROS-related mitochondrial dysfunction in skeletal muscle of an ALS mouse model during the disease progression. Pharmacol. Res..

[5] Forostyak S (2014). Intrathecal delivery of mesenchymal stromal cells protects the structure of altered perineuronal nets in SOD1 rats and amends the course of ALS. Stem Cell.

[6] Mancuso R (2014). Resveratrol improves motoneuron function and extends survival in SOD1(G93A) ALS mice. Neurotherapeutics.

[7] Oh KW (2018). Repeated Intrathecal Mesenchymal Stem Cells for Amyotrophic Lateral Sclerosis. Ann. Neurol..

[8] Peters, S. *et al*. The TgF-beta system as a Potential Pathogenic Player in Disease Modulation of amyotrophic lateral sclerosis. *Front Neurol*, **8**, 10.3389/fneur.2017.00669 (2017). 10.3389/fneur.2017.00669 PMC5736544 29326641

[9] Gonzalez, D. *et al*. ALS skeletal muscle shows enhanced TGF-beta signaling, fibrosis and induction of fibro/adipogenic progenitor markers. *Plos One*, **12**, 10.1371/journal.pone.0177649 (2017). 10.1371/journal.pone.0177649 PMC5433732 28520806

[10] Si, Y. *et al*. Transforming Growth Factor Beta (TGF-beta) Is a Muscle Biomarker of Disease Progression in ALS and Correlates with Smad Expression. *Plos One*, **10**, 10.1371/journal.pone.0138425 (2015). 10.1371/journal.pone.0138425 PMC4574401 26375954

[11] Abrigo J, Rivera JC, Simon F, Cabrera D, Cabello-Verrugio C (2016). Transforming growth factor type beta (TGF-beta) requires reactive oxygen species to induce skeletal muscle atrophy. Cell Signal..

[12] Lim MA (2012). Reduced activity of AMP-activated protein kinase protects against genetic models of motor neuron disease. J. Neurosci..

[13] Perera, N. D. *et al*. Mutant TDP-43 Deregulates AMPK Activation by PP2A in ALS Models. *Plos One*, **9**, 10.1371/journal.pone.0090449 (2014). 10.1371/journal.pone.0090449 PMC3942426 24595038

[14] Liu YJ, Lee LM, Lai HL, Chern Y (2015). Aberrant activation of AMP-activated protein kinase contributes to the abnormal distribution of HuR in amyotrophic lateral sclerosis. FEBS Lett..

[15] Liu YJ (2015). Activation of AMP-activated protein kinase alpha1 mediates mislocalization of TDP-43 in amyotrophic lateral sclerosis. Hum. Mol. Genet..

[16] Kaneb, H. M., Sharp, P. S., Rahmani-Kondori, N. & Wells, D. J. Metformin Treatment Has No Beneficial Effect in a Dose-Response Survival Study in the SOD1(G93A) Mouse Model of ALS and Is Harmful in Female Mice. *Plos One*, **6**, 10.1371/journal.pone.0024189 (2011). 10.1371/journal.pone.0024189 PMC3164704 21909419

[17] Coughlan KS, Mitchem MR, Hogg MC, Prehn JHM (2015). “Preconditioning” with latrepirdine, an adenosine 5 ‘-monophosphate-activated protein kinase activator, delays amyotrophic lateral sclerosis progression in SOD1(G93A) mice. Neurobiol. Aging.

[18] Samuel MA (2014). LKB1 and AMPK regulate synaptic remodeling in old age. Nat. Neurosci..

[19] Dial AG, Ng SY, Manta A, Ljubicic V (2018). The Role of AMPK in Neuromuscular Biology and Disease. Trends Endocrinol. Metab..

[20] Gugliandolo A, Bramanti P, Mazzon E (2019). Mesenchymal Stem Cells: A Potential Therapeutic Approach for Amyotrophic Lateral Sclerosis?. Stem Cell Int..

[21] Rehorova, M. *et al*. A Combination of Intrathecal and Intramuscular Application of Human Mesenchymal Stem Cells Partly Reduces the Activation of Necroptosis in the Spinal Cord of SOD1(G93A) Rats. *Stem Cells Transl Med*, 10.1002/sctm.18-0223 (2019). 10.1002/sctm.18-0223 PMC6525562 30802001

[22] Petrou P (2016). Safety and Clinical Effects of Mesenchymal Stem Cells Secreting Neurotrophic Factor Transplantation in Patients With Amyotrophic Lateral Sclerosis: Results of Phase 1/2 and 2a Clinical Trials. JAMA Neurol..

[23] Sykova E (2017). Transplantation of Mesenchymal Stromal Cells in Patients With Amyotrophic Lateral Sclerosis: Results of Phase I/IIa Clinical Trial. Cell Transpl..

[24] Abrigo J, Simon F, Cabrera D, Cabello-Verrugio C (2016). Angiotensin-(1-7) Prevents Skeletal Muscle Atrophy Induced by Transforming Growth Factor Type Beta (TGF-beta) via Mas Receptor Activation. Cell Physiol. Biochem..

[25] Park CM (2016). Umbilical cord mesenchymal stem cell-conditioned media prevent muscle atrophy by suppressing muscle atrophy-related proteins and ROS generation. Vitro Cell Dev. Biol. Anim..

[26] Sriramulu S (2018). Concise Review on Clinical Applications of Conditioned Medium Derived from Human Umbilical Cord-Mesenchymal Stem Cells (UC-MSCs). Int. J. Hematol. Oncol. Stem Cell Res..

[27] Bak DH (2019). Antioxidant effect of human placenta hydrolysate against oxidative stress on muscle atrophy. J. Cell Physiol..

[28] Abrigo J (2018). Role of Oxidative Stress as Key Regulator of Muscle Wasting during Cachexia. Oxid. Med. Cell Longev..

[29] Pollari E, Goldsteins G, Bart G, Koistinaho J, Giniatullin R (2014). The role of oxidative stress in degeneration of the neuromuscular junction in amyotrophic lateral sclerosis. Front. Cell Neurosci..

[30] Ichim TE (2010). Mesenchymal stem cells as anti-inflammatories: implications for treatment of Duchenne muscular dystrophy. Cell Immunol..

[31] Suzuki M (2008). Direct muscle delivery of GDNF with human mesenchymal stem cells improves motor neuron survival and function in a rat model of familial ALS. Mol. Ther..

[32] Hall, D. T. *et al*. The AMPK agonist 5-aminoimidazole-4-carboxamide ribonucleotide (AICAR), but not metformin, prevents inflammation-associated cachectic muscle wasting. *Embo Molecular Medicine*, 10, 10.15252/emmm.201708307 (2018). 10.15252/emmm.201708307 PMC6034131 29844217

[33] Ma JF (2017). STAT3 promotes IFNgamma/TNFalpha-induced muscle wasting in an NF-kappaB-dependent and IL-6-independent manner. EMBO Mol. Med..

[34] Cramer, Z. *et al*. eIF4A inhibition prevents the onset of cytokine-induced muscle wasting by blocking the STAT3 and iNOS pathways. *Scientific Reports*, **8**, 10.1038/s41598-018-26625-9 (2018). 10.1038/s41598-018-26625-9 PMC5976662 29849089

[35] Powers SK, Ji LL, Kavazis AN, Jackson MJ (2011). Reactive oxygen species: impact on skeletal muscle. Compr. Physiol..

[36] Schieber M, Chandel NS (2014). ROS function in redox signaling and oxidative stress. Curr. Biol..

[37] Finkel T, Holbrook NJ (2000). Oxidants, oxidative stress and the biology of ageing. Nat..

[38] Ragusa RJ, Chow CK, Porter JD (1997). Oxidative stress as a potential pathogenic mechanism in an animal model of Duchenne muscular dystrophy. Neuromuscul. Disord..

[39] Kook MG (2017). KCHO-1, a novel herbal anti-inflammatory compound, attenuates oxidative stress in an animal model of amyotrophic lateral sclerosis. J. Vet. Sci..

[40] Seo Y (2011). Human umbilical cord blood-derived mesenchymal stem cells protect against neuronal cell death and ameliorate motor deficits in Niemann Pick type C1 mice. Cell Transpl..

[41] Shin TH (2016). Human umbilical cord blood-stem cells direct macrophage polarization and block inflammasome activation to alleviate rheumatoid arthritis. Cell Death Dis..

