# Supplementary material for: Repeated intramuscular transplantations of hUCB-MSCs improves motor function and survival in the SOD1 G93A mice through activation of AMPK
Source: Sci Rep. 2020 Jan 31;10:1572. doi: 10.1038/s41598-020-58221-1 (PMC6994691; doi:10.1038/s41598-020-58221-1)
Supplement: Supplementary file 1 — Supplementary information . [file 41598_2020_58221_MOESM1_ESM.pdf]

# Repeated intramuscular transplantations of hUCB-MSCs improves motor function and survival in the SOD1 $G^{93}A$ mice through activation of AMPK.

**Myung Geun Kook[1,2], SeungEun Lee[1,2], Nari Shin[1,2], Dasom Kong[1,2], Da-Hyun Kim[1,2], Min-Soo Kim[1,2], Hyun Kyoung Kang[1,2], Soon Won Choi[1,2] & Kyung-Sun Kang[1,2*]**

[1]Adult Stem Cell Research Center, College of Veterinary Medicine, Seoul National University, Seoul 08826, Republic of Korea. [2]College of Veterinary Medicine and Research Institute for Veterinary Science, Seoul National University, Seoul 08826, Republic of Korea. Correspondence and requests for materials should be addressed to K.-S.K. (emails: kangpub@snu.ac.kr )

## Supplement Figure

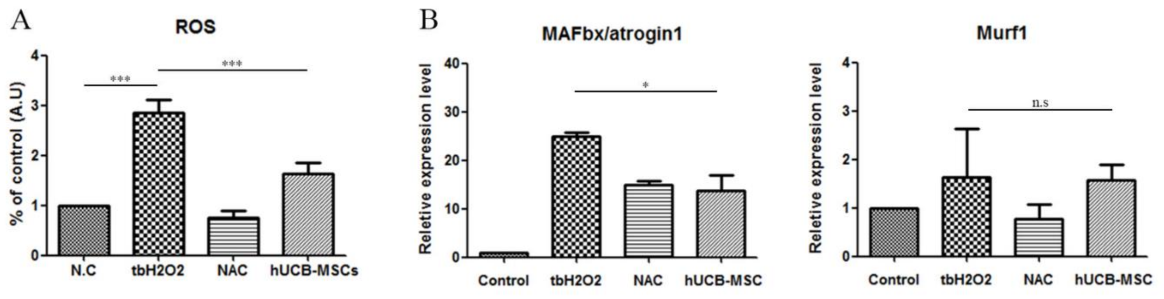


Supplementary Figure S1. hUCB-MSCs prevented muscle atrophy and reduced ROS levels in C2C12 cells. (A) hUCB-MSCs reduced generation of intracellular hydrogen peroxide induced by $tbH_2O_2$ in C2C12 myoblast cells. (B) hUCB-MSCs significantly prevented MAFbx/atrogin1 mRNA expression, but not Murf1 mRNA expression. All data represented are means ± SEM and analyzed by one way analysis. *P<0.05, **P<0.01, ***P<0.001

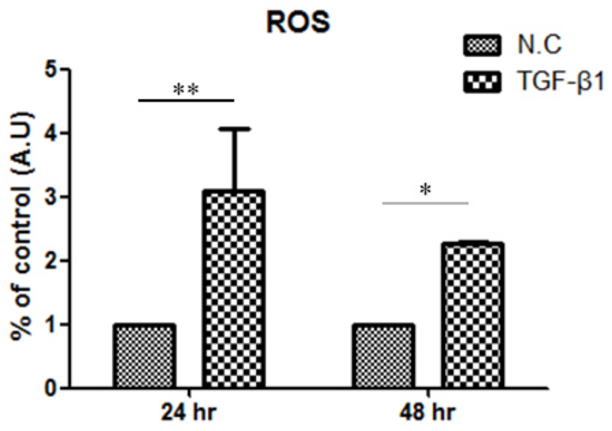


Supplementary Figure S2. TGF-β1 significantly increased ROS level at 24 hr.

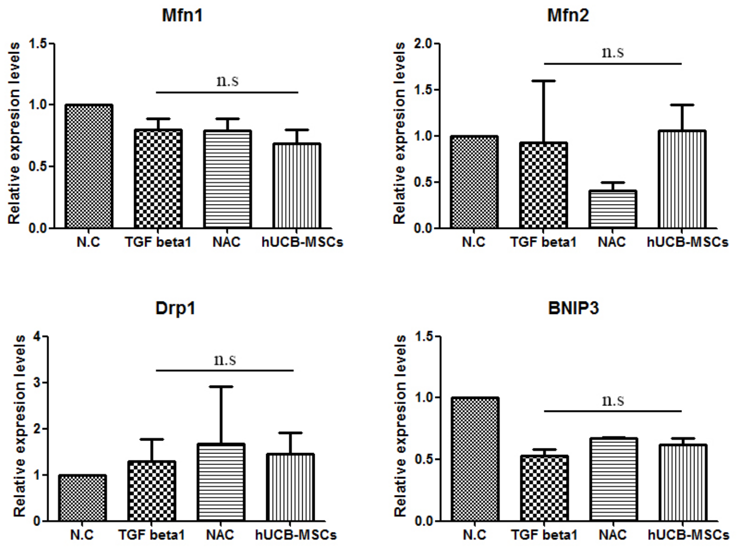


Supplementary Figure S3. TGF-β1 and hUCB-MSCs did not altered mitochondrial related genes in C2C12 cell myotubes. Transcript levels of mitochondrial related genes were determined by quantitative real-time PCR. Transcript levels of each genes were not significantly altered.

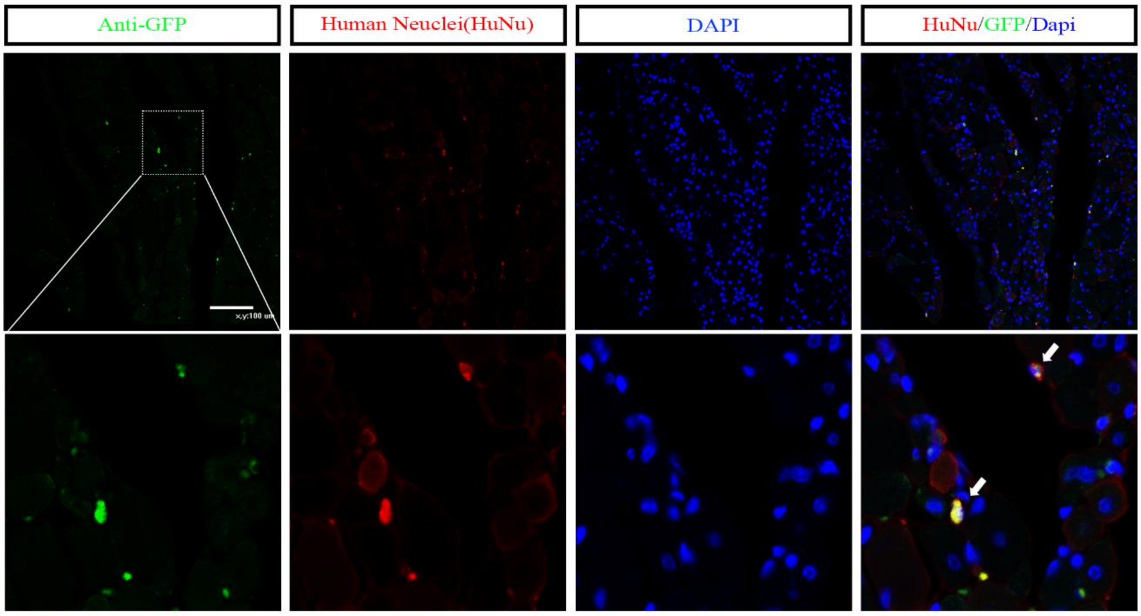


Supplementary Figure S4. Survival of hUCB-MSCs in the gastrocnemius muscle. For investigating survival of hUCB-MSCs, GFP tagged hUCB-MSCs were used. One week after transplantation of hUCB-MSCs in gastrocnemius muscle, human specific markers for nuclei (HuNu) and anti-GFP expression were detected.

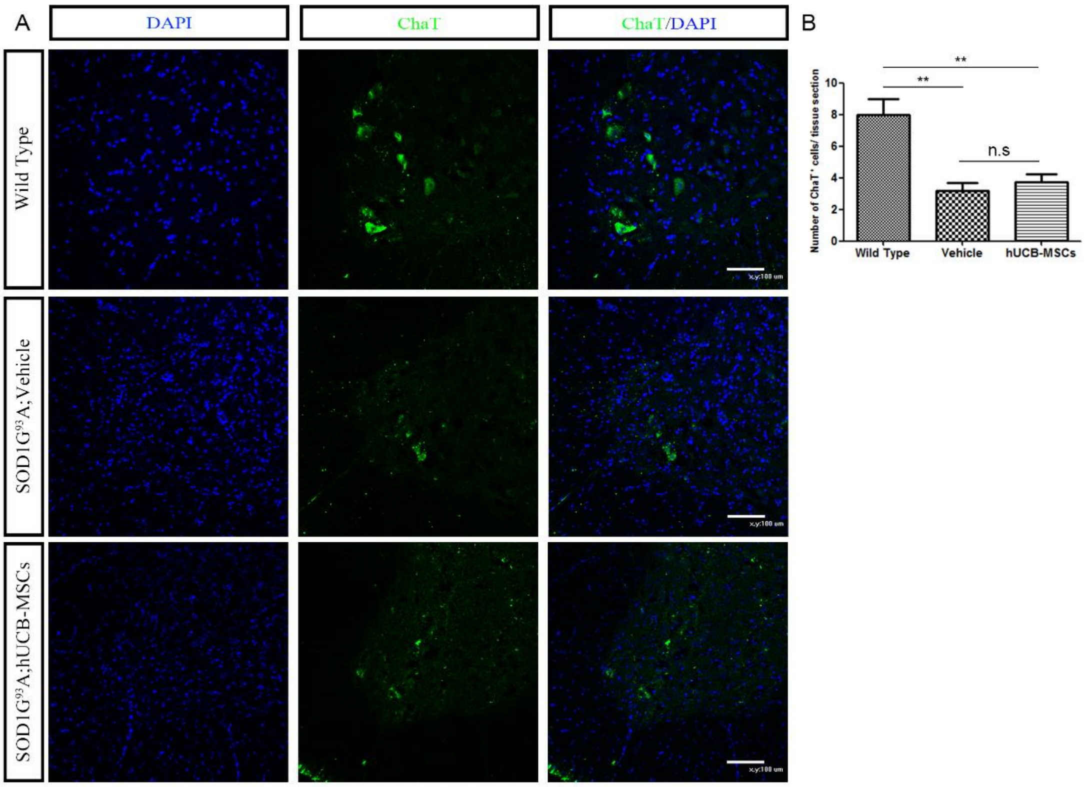


Supplementary Figure S5. Repeated intramuscular transplantation of hUCB-MSCs did not prolong survival of the motor neurons in the spinal cord of SOD1 transgenic mice. (A) Representative images of chat positive motor neurons in the spinal cord of SOD1 transgenic mice after application of vehicle and hUCB-MSCs. (B) The motor neuron loss was significantly increased in SOD1 transgenic mice but in vehicle and hUCB-MSCs groups the number of motor neurons was not significantly different. All data represented are means ± SEM and analyzed by one way analysis. **P<0.01

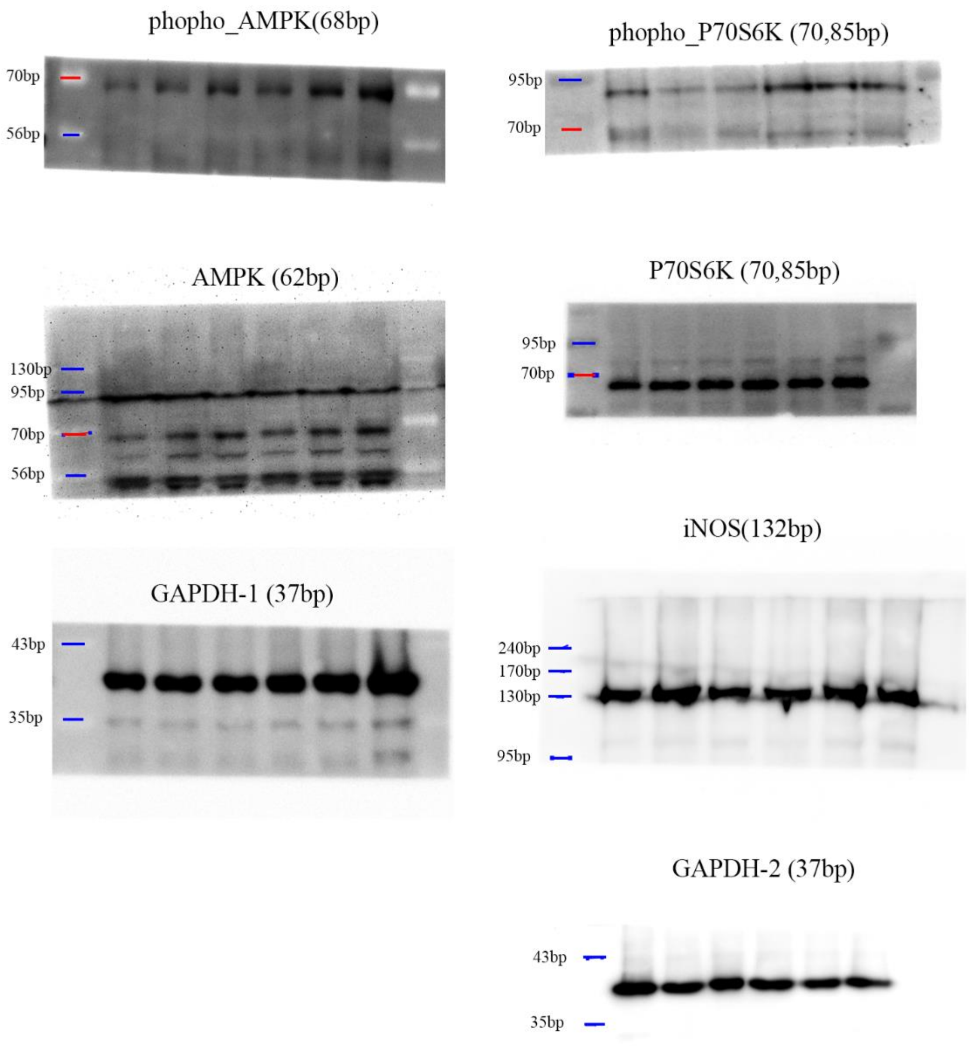


Supplementary dataset1 (Figure 3)

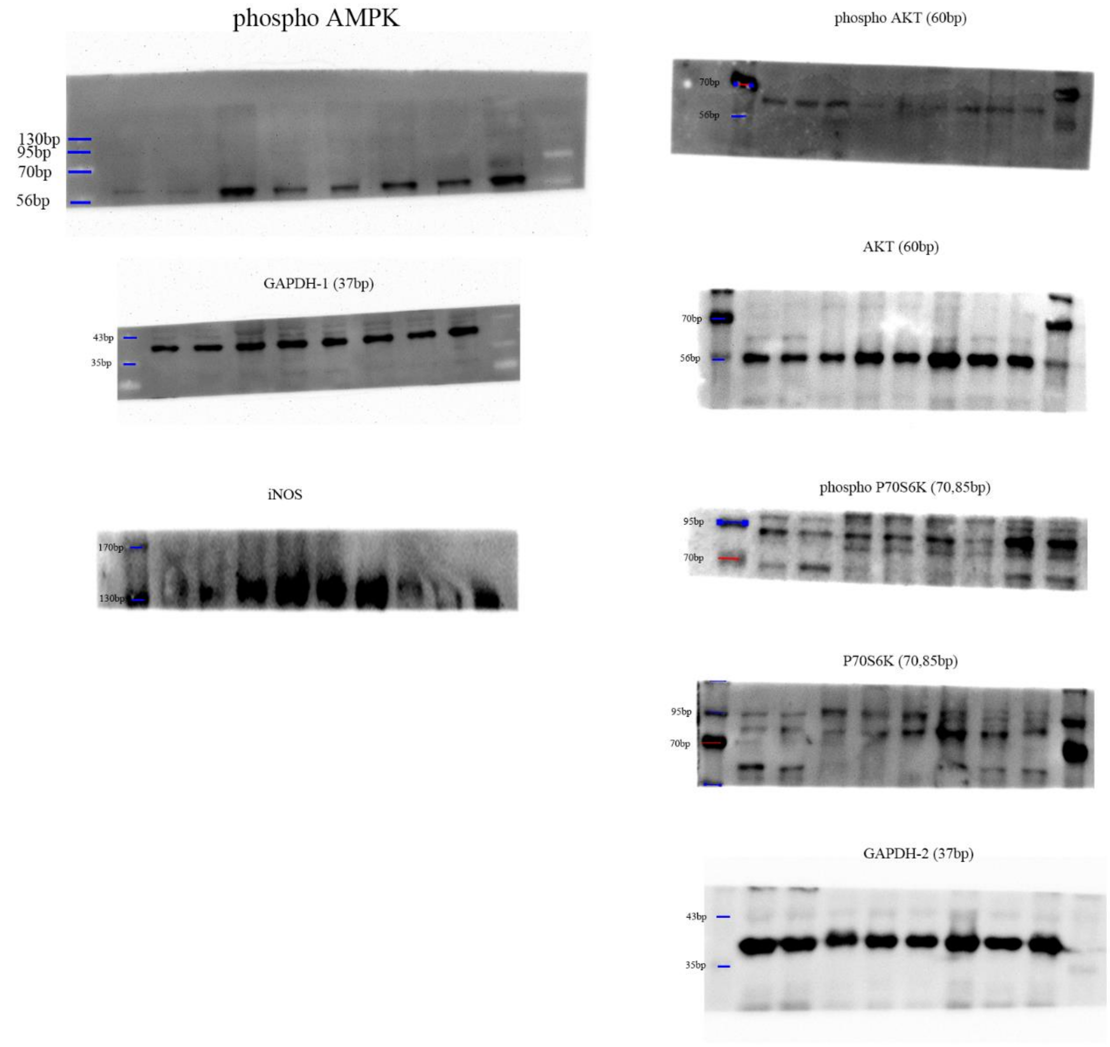


Supplementary dataset2 (Figure 6)
